# Cash transfers for HIV prevention: what do young women spend it on? Mixed methods findings from HPTN 068

**DOI:** 10.1186/s12889-017-4513-3

**Published:** 2017-07-11

**Authors:** Catherine MacPhail, Nomhle Khoza, Amanda Selin, Aimée Julien, Rhian Twine, Ryan G. Wagner, Xavier Goméz-Olivé, Kathy Kahn, Jing Wang, Audrey Pettifor

**Affiliations:** 1 School of Health and Society, University of Wollongong, Wollongong, NSW Australia; 2 Wits Reproductive Health and HIV Institute, University of the Witwatersrand, Johannesburg, South Africa; 3 Carolina Population Center, University of North Carolina, Chapel Hill, NC USA; 4 MRC/Wits Rural Public Health and Health Transitions Research Unit, School of Public Health, Faculty of Health Sciences, University of the Witwatersrand, Johannesburg, South Africa; 5 INDEPTH Network, Accra, Ghana; 6 Umeå Centre for Global Health Research, Umeå, Sweden; 7 Fred Hutchinson Cancer Research Center, Seattle, WA USA; 8 Department of Epidemiology, University of North Carolina, Chapel Hill, NC USA

**Keywords:** Cash transfers, Adolescents, Spending, HIV prevention

## Abstract

**Background:**

Social grants have been found to have an impact on health and wellbeing in multiple settings. Who receives the grant, however, has been the subject of discussion with regards to how the money is spent and who benefits from the grant.

**Methods:**

Using survey data from 1214 young women who were in the intervention arm and completed at least one annual visit in the HPTN 068 trial, and qualitative interview data from a subset of 38 participants, we examined spending of a cash transfer provided to young women conditioned on school attendance.

**Results:**

We found that spending was largely determined and controlled by young women themselves and that the cash transfer was predominately spent on toiletries, clothing and school supplies. In interview data, young women discussed the significant role of cash transfers for adolescent identity, specifically with regard to independence from family and status within the peer network. There were almost no negative consequences from receiving the cash transfer.

**Conclusions:**

We established that providing adolescents access to cash was not reported to be associated with social harms or negative consequences. Rather, spending of the cash facilitated appropriate adolescent developmental behaviours. The findings are encouraging at a time in which there is global interest in addressing the structural drivers of HIV risk, such as poverty, for young women.

**Trial registration:**

Clinicaltrials.gov NCT01233531 (1 Nov 2010). First participant enrolled 5 March 2011.

## Background

Social grants have been found to impact health and wellbeing in multiple settings [1]. Worldwide, social grants have traditionally been paid to adult women [2]. There is evidence showing that cash transfers paid to adult women are more likely to be used for the good of the household [3] than those given to men, which might be used for socially irresponsible behaviors with negative outcomes [4]. Who receives the grant, however, has been the subject of discussion with regards to how grant money is spent and who benefits from the grant. There are concerns that cash to adolescents might be more vulnerable than adults to social pressures, with the overriding concern being that providing access to cash to adolescents by making social grant payments to them might result in undesirable behaviors or place adolescents at risk of social harms. Such concerns are particularly significant in South Africa where a large-scale government social protection program has provided grants to caregivers of children in poor households since 1998. From the inception of the Child Support Grant (CSG), there have been fears about adolescents deliberately getting pregnant to access resources through the CSG [5, 6]. Rumors of the role of the CSG in promoting teenage pregnancies abound in the media and community consciousness, despite a number of studies establishing that there is no such relationship [7–9]. Such concerns are in the context of a growing materialism in South African society [10] and evidence from other contexts of the key symbolic value of consumer goods for positioning young people in their social networks and the development of their identities [11, 12].

A recent focus on the potential for social protection programs to limit HIV risk has led to an increase in HIV prevention programs adopting a social protection approach. However, relatively few pay cash transfers directly to adolescents [13–15]. One of the first to pay young women was in rural Zomba province of Malawi. The project implemented both conditional (school attendance) and unconditional payments ranging from USD1 to USD5, as well as making small payments to parents [13]. In South Africa conditional payments were paid to young people in KwaZulu-Natal province for a range of behaviors, including school attendance [16]; and an ongoing combination HIV prevention intervention is paying cash transfers to female adolescent in Tanzania [17]. However, there has been little investigation of how cash transfers are spent by adolescents; whether concerns about access to cash promoting undesirable behaviors are warranted; and limited discussion of the meaning that access to such money might have for adolescents in impoverished environments. Using mixed methods data from the HPTN 068 trial of a conditional cash transfer for HIV prevention in South Africa, we examine young women’s cash transfer spending patterns and social impacts that access to cash engendered.

### The Swa Koteka (HPTN 068) cash transfer project

This analysis was situated within the Swa Koteka cash transfer project (HPTN 068). The study was a phase III individually randomized trial to assess the impact of a cash transfer conditioned on school attendance on HIV and HSV-2 incidence and other behavioral outcomes [18]. The study provided young women and their parent/guardian in the intervention arm with a monthly cash transfer conditional on 80% monthly school attendance. Young women and their parent/guardian in the control arm did not receive any payment. The study was conducted in the Medical Research Council/Wits University Agincourt Health and Socio-Demographic Surveillance System (HDSS) site in the rural Bushbuckridge sub-district of Mpumalanga Province in South Africa. The area currently covers approximately 450 km^2^, contains 32 villages and has largely underdeveloped infrastructure. The majority of roads are unpaved and commercial and retail space is limited. In this area, there are high levels of unemployment and poverty, and strong reliance on government social grants. HIV prevalence in the area is high. During 2011, HIV prevalence was 5.5% among 15–19 year old women and increased to 46% for women aged 35–39 years [19].

At enrollment into the study, young women and their parent/guardian in the intervention arm were asked to open a South African post office banking account if they did not already have a bank account. This was the preferred approach to making payments to young women for a number of reasons. There are two post offices located in the study site and one in a township close by; the documentation requirements for opening an account are less stringent than for banks, significant for some families who have not accessed birth and other registration documents; and unlike banks, there were no administrative charges on the accounts. Additionally, the study site has limited Automated Teller Machine (ATM) banking infrastructure. There were no ATMs in the area at the start of the study, although over the duration of the study ATMs became available in the center of the site, and an unknown number of cash points at various smaller shops emerged, owing to an initiative for more access from a major bank. Young women therefore had to travel to access their cash. The project was not able to make payments in cash, as has been the case in other similar studies [20], because we had no means to ensure staff and young women’s safety when carrying large amounts of cash. Instead, we used automated electronic payments to make monthly deposits into young women’s accounts. Total monthly payments of ZAR300 were made through the study (ZAR100 to young women themselves and ZAR200 to their nominated parent or guardian – approximately USD12 and USD24, respectively). This amount was matched to CSG payments at the time that the study started. Additional information on how we assessed eligibility for monthly payments based on recorded school attendance is available elsewhere [18].

During HPTN 068 we collected both quantitative and qualitative data on the spending patterns of women in the intervention arm and their parents. In this analysis we focus only on young women’s spending to explain the management of money and spending patterns of young women in South Africa who receive cash transfers. Moreover, we reflect on concerns about the belief that payment of cash transfers to adolescents might lead to negative consequences and harms.

## Methods

### Survey data

Study participants were drawn from a sample of young women aged 13–20 years enrolled in schools within the Agincourt HDSS. Inclusion criteria were that young women should not be pregnant or married at baseline; they and their parent/guardian should have the necessary documentation to open a bank account; and they should be currently residing in the study area and attending a local school. After obtaining written informed consent from a parent/guardian and written informed consent or assent from the young women, all participants completed a baseline interview using Audio Computer Assisted Self Interview (ACASI), provided blood samples for HIV and HSV-2 testing and were randomized to receive the cash transfer or not. Young women were then interviewed annually until the study completed or until they graduated from high school, whichever happened first. Each annual visit included an ACASI interview, blood draw, HIV and HSV-2 testing for those negative at their previous visit, and HIV and HSV-2 counseling. Full details of the trial methods and outcomes are available elsewhere [21]. Surveys were also completed by parent/guardians; however, this analysis is limited to young women’s data.

### Qualitative cohort

A sub-sample of young women was recruited into a longitudinal qualitative cohort with whom repeated interviews were conducted in order to explore experience over time [22]. Purposive sampling was used to ensure that qualitative data overrepresented intervention participants, sexually active young women, girls who reported missing school and those reporting transactional sex. This was to ensure that issues we hypothesized would be relevant to experience of the cash transfer would be represented. Participants were informed that the study team was interested in talking to young women about their experiences of the intervention and thoughts about the cash transfers. They were also informed that they would be asked to reflect on their attitudes and beliefs about schooling, sex and HIV risk for young people in South Africa. Participants provided additional written informed consent for participation in the qualitative cohort at the start of the first interview and this was verbally reconfirmed before each subsequent interview.

Semi-structured interviews were conducted twice annually throughout young women’s engagement in the trial for a potential total of six interviews per participant. As young women in the qualitative cohort exited the main study, they were replaced by young women with similar characteristics; data is therefore largely from women over 15 years of age, although many had been enrolled in the trial from 13 to 15 years old. A total of 39 intervention young women were recruited into the qualitative cohort. Face-to-face interviews of between 30 and 90 min were conducted by a team of four experienced qualitative interviewers. The interviewers were all female, local residents with a minimum grade 12 completed education and fluent xiTsonga speakers. We conducted refresher training in qualitative interviewing at the start of the study and at regular intervals throughout the duration of the data collection. We also conducted topic guide specific training at the start of each round of qualitative data collection. Training focused on the meaning and intentions of questions in the interview guide, appropriate xiTsonga language for the interview concepts and role playing to develop familiarity with questions and procedures. Each participant was allocated to a single interviewer to ensure that they developed a relationship over time and so that interviewers could reflect on information from previous interviews. The interviewer reviewed previous interview transcripts prior to each subsequent interview to improve their recall of information previously provided by participants.

At each interview, young women were asked a range of questions about how they were managing the cash transfer. This included questions such as ‘How do you usually spend your money?’, ‘How do you feel about having access to your own money?’, ‘Tell me about any experiences you have had with someone taking your Swa Koteka money from you.’ We also asked about negative and positive experiences and feelings associated with receiving the cash transfer. We asked about the impact of receiving the grant on relationships within the family and community through questions such as ‘Have there been any negative outcomes in your family after receiving the money?’ after which we probed to establish what these negative outcomes were (for example, arguments in the household about controlling or spending the cash transfer). Additional probes were provided for the interviewers to use for all questions, but they were also encouraged to use probes that best suited the context of each interview.

### Data analysis

Qualitative interviews were digitally recorded and then translated and transcribed in a single step by the person who conducted the interview. All transcripts were checked for clarity and then coded in Atlas.ti (version 7) making use of a coding frame that was both inductively and deductively generated by two of the authors (CM and NK) following a framework approach [23]. In this approach themes are generated from a priori issues and questions guiding the objectives and aims of the study (induction), as well as issues generated from the data (deduction). Coding was conducted by five researchers who completed initial training and worked towards acceptable intercoder reliability (ICR) scores on initial study transcripts [24]. A random sample of 10% of the transcripts was double coded throughout the study to ensure that ICR remained acceptable.

For analysis of the qualitative data, codes directly linked to the theme of ‘spending’ and ‘grant experience’ were examined. These codes included information about items on which young women spent their portion of the cash transfer, discussions about saving the transfer, their experience of having access to their own money and how this money was controlled within the family. We also examined codes that we had used to document positive and negative outcomes of receiving the cash transfer.

We analyzed quantitative data only from baseline HIV-negative intervention young women who completed at least one follow up visit (*n* = 1209). Within this sample the median visit number was two. For this analysis, we focused on the module of the questionnaire that asked about cash transfer spending habits. At each annual follow-up visit we asked intervention young women a range of questions about their understanding of the cash transfer and their experience of both accessing and spending the transfer. To assess spending of the cash transfer we analyzed the question ‘What was the Swa Koteka transfer allocated to you spent on?’ To examine who controlled the young women’s portion of the cash transfer we descriptively analyzed a range of questions including: ‘Who usually spends your portion of the Swa Koteka money?’ and ‘Who decides how to spend the Swa Koteka money allocated to you?’ Finally, we examined data from the question ‘Who normally withdraws your R100 Swa Koteka money?’ Each category of spending question was summarized across all young women and all data collection points. The records with missing responses to the question were excluded from the analysis. Across all 2597 visits, 31 records were missing for spending questions and 45 were missing for other questions. Logistic regression with robust variance to account for repeated measurement on each young woman was used to test the association between demographic variables and young women’s decisions about managing the cash transfer.

During analysis, data was triangulated through comparison of the findings from each of the data sources. Explanations for behaviors reported in the quantitative survey were illustrated with participants’ words in the qualitative data and differences between the two sources specifically noted.

## Results

### Decision making about spending the cash transfer

From the **quantitative survey** data it was clear that decision making about spending the cash transfer rested overwhelmingly with the young women themselves. Across all data collection points, 78% percent of responses indicated that decisions about spending the money were their own; a further 18% indicated that an adult or other household member told them how to spend the money and 3% percent discussed how the money should be spent with other household members. A very small percentage (<1%) indicated that their boyfriends had a role in decision making about the cash transfer. This level of autonomy is further reflected in who physically accessed the money and spent it. Banking infrastructure is limited in the study site, with post offices in two villages at the start of the study and ATMs/cash points becoming more available across the site over the duration of the study. Two-thirds of responses indicated that young women accessed their money from the post office (rather than a bank ATM), largely in the study site. A further 20% of responses indicated travelling between 30 min to 1 h, depending on their location in the study site, to a nearby larger town to withdraw their money. The remainder (13%) indicated travelling to other (unspecified) places to withdraw their cash transfer. Despite the necessity for many participants to travel to access the money, 73% of responses indicated that young women withdrew the cash themselves, followed by mothers (15.4%). Involving other family or household members in withdrawing the money was negligible (less than 3% for all categories), but specifically involved other female relatives such as older sisters and grandmothers. Older participants were significantly more likely to report withdrawing the cash themselves (*p* < 0.0001) when comparing younger women (13–16 years) to older women (17 years and older).

Young women’s autonomy in accessing their cash transfers was echoed in actual spending of their portion of the cash transfer, with 80% indicating that they spend the money themselves. Approximately 10 % of participants reported that their mothers also spent their portion of the cash transfer, but less than 2 % reported other family members spending their money.

In **qualitative discussions**, there were very few reports of young women having their money forcefully taken or being sidelined in decision making about spending the money. In one instance, a young woman hints that this has been attempted, when she says ‘*they do not take it. I resist’,* although she did not discuss this issue further in subsequent discussions*.* Another young woman spoke about giving her money to her mother for safe keeping and to enforce some degree of saving. She noted that her mother was completely trustworthy in this regard but then mentioned a friend, who is also a study participant, and whose mother cannot be trusted because she does not return money given to her for safe keeping.

*She doesn’t give the money to her Mom because her Mom will use the money and she won’t give it back to her. So she keeps it, but it won’t last for a week because she buys her friends things and she ends up not knowing what she spent her money on.* (Participant 5956, 19 years, Interview 1/1)^1^

Similarly, a 19-year-old participant spoke during her interviews of her family’s declining relationship with her step-father. She discussed how there were complaints in the household about the cost of food and that her mother and step-father were *‘saying that I must contribute in the household.’* In a context of general concern about household resources, her control of the cash transfer became contested and there were instances in which her money was used without her consent.

*I’m not the one who drew my last payment. My mother has taken it and said that she bought relish. I didn’t say anything. She said that she’s only left with R50 then I said to her ‘Please keep it for me. I want to buy some shoes.’ Last Monday I was in need of money, then I requested R50 from my mother but she said that she had given it to my stepfather to use it on transport. With this money I didn’t say anything until now. She said he will come back on Friday. I don’t know if he will give her the money or not. If he doesn’t give my mother I will accept it no problem.* (Participant 4728, 19 years, Interview 2/3)

Finally, there was one report of the cash transfer being stolen. The young woman mentioned that both her identification document and post bank card were stolen, and her belief that someone in her family was responsible: *‘I can say that it is a family member because there is no one who knows here better than family members.’* There were no other reports of negative outcomes related to accessing the cash transfer. This in keeping with our assessment of social harms during the study (*n* = 16), the majority of which were related to teasing for study participation and reported by both intervention and control participants [21].

### Items bought with the cash transfer

In the **quantitative survey** data young women reported spending their cash transfers on a range of items. The most common item was toiletries (56.2%), followed by clothing (35.0%), school uniform and school supplies (30.5%), mobile phones, ringtones and airtime (29.7%), hairdressing (29.3%), cool drinks, sweets and fast foods (25.6%), and makeup or cosmetics (20.5%). We noted a difference in spending patterns as participants aged within the study. There was a significant difference (*p* < 0.05) in spending between older participants (17 years and older) and younger participants (13–16 years) on toiletries, mobile phones, makeup/cosmetics, hairdressing, school uniform or supplies, birth control, transportation, health care and child care. Responses to these items were more common among the older participants.

There was little reporting of spending on items perceived to be detrimental to young women’s health and wellbeing. Only 1.8% reported buying cigarettes and 2.3% reported using the money to buy alcohol. Echoing data reported in the surveys, in the **qualitative interviews** young women talked about items that were viewed as irresponsible spending and for which they did not use the cash transfer.

*There are no risk behaviors because of this money. I know how to manage it. I don’t buy drugs or alcohol, most of the time I spend the money on the most important things. I don’t abuse the money.* (Participant 6961, 17 years, Interview 1/5)

*If someone can buy for me, I drink. But I cannot take my money and buy alcohol.* (Participant 1343, 18 years, Interview 4/5)

In discussions with young women there was a sense that the money needed to be spent ‘responsibly’; this perception appeared to stem from conditioning cash payments on school attendance, although decisions about spending the cash transfer were not prescribed or discussed with participants.

Given that the payment was conditioned on school attendance, it seems that many participants and their families viewed the money as being intended for school related items such as school uniforms and stationary supplies.

*Everything that Swa Koteka study has brought in the community is good. Because the study wants us to focus on our education and be able to take care of ourselves.* (Participant 8254, 18 years, Interview 2/2)

Spending that might be related to the outcome of interest in the main study (HIV incidence) was low, with only 9.25% mentioning that they used the money to purchase birth control or condoms (grouped in the analysis).

Similarly, despite the relative poverty of the area in which the young women live, the young women’s portion of the cash transfer was rarely used to support the household more generally. Less than 10% of young women reported that their portion of the cash transfer was used for general household groceries (8.8%) or paying rent and utilities (2.1%). When asked specifically to comment on whether their portion of the cash transfer was used for groceries, one participant responded that *‘the one for the parent we buy food’*, reflecting the general belief that the young women’s portion was not intended for general household spending. Despite some participants already having children at baseline and pregnancies in the cohort subsequent to study enrollment, only 1.9% of intervention girls reported spending their cash transfer on crèche or childcare. Given that the intervention only recruited young women who were already enrolled in school, this might mean that financing childcare arrangements had already been made to accommodate their schooling, such as applying for and receiving a CSG for their child.

Triangulation of the quantitative and qualitative data from the study validated the information on spending. Reports across the interviews matched the items that young women reported spending money on in the quantitative surveys, with one significant addition. In the interviews, young women were most likely to mention taking the money to school as ‘pocket money’ and sharing their money with siblings for this purpose. This particular category was not provided to young women in the quantitative survey, although is likely represented in the 25.6% who reported spending the cash transfer on cool drinks, chips, sweets or fast foods. Across the qualitative interviews, there were few instances in which participants discussed spending their cash transfer on more substantial items, with two notable exceptions. One recognized that she was losing R20 each time she had to pay for transport to get to the ATM to withdraw her money and therefore started a small business selling sweets to recoup this money.

*I have a small business. I am selling some sweets. . . . I started my business since I started to get the money from Swa Koteka. I am trying to make extra income and I am getting profit. The reason why I started this business is that I want to get the money I pay for transport when I go to Thulamahashe to withdraw the Swa Koteka money.* (Participant 5833, 17 years, Interview 1/3)

Another decided that having an identification document was significant for her future and determined to use the cash transfer payment to get this vital document. She noted:

*I decided to spend the money on the ID document when the principal announced at school that there were people from [the Department of] Home Affairs who will come at school to assist us register for ID documents. So I regarded it as an important thing to me since I told you that I usually spend the money on the most important things.* (Participant 6961, 15 years, Interview 1/5)

We did not ask young women in the quantitative component of the study whether they saved the cash transfer specifically. This was however reported by very few young women in qualitative interviews; mostly reporting that they asked their mothers to keep the money for them so that they were not tempted to spend it on smaller items. One participant noted that *‘she [mother] deposits the money into her bank account and then I will take the money when it’s R1000.’* Such forms of saving were generally short term and with specific purchases in mind, such as clothes for the Christmas season or a particular occasion. A very small number of young women reported that they were saving their money for longer term goals such as university costs. One participant consistently saved her cash transfer through the life of the project, but another, who began the study saving for university, admitted in subsequent interviews that she had stopped saving as *‘I’m a human being and I have needs.’* Another young woman discussed her plans to begin saving, but acknowledged that she had not yet started.

*I haven’t started saving the money but I’m thinking of it. I don’t know if I can be able to do it, but I think of saving the money. I will take the R200 that my mother gets and that R100 for me and save it into one account so that I can use it when I go to tertiary school [university]. I will apply for a bursary, and then I will use the money for food and paying for accommodation.* (Participant 3285, 18 years, Interview 1/4)

Although there was generally an acceptance that the cash transfer amount was appropriate, the quote above is indicative of the fact that most participants reported spending most or all of their cash transfer every month. While saving for a long-term future might have been desired by participants, it was not something that most felt was practically possible in their current circumstances.

### Sharing the money

In most instances there was no expectation that young women would share their portion of the cash transfer with other members of their household. Indeed, in some cases parents chose to give the household portion of the cash transfer to the young woman as well as their own portion. In the qualitative interviews most young women noted that they didn’t share the cash transfer, with one participant noting, *‘no I didn’t give it to anyone and I like money too much. I can’t survive without money’.* Despite there being no expectation that young women would share their portion of the cash transfer, many chose to do so anyway. One of the participants talked about sharing widely in the family, saying: *‘If my sibling asks for money I give him, my mom too. If she asks for money, I give her. Sometimes she borrows my money.’* Sharing was most often to provide younger siblings with pocket money at school or particularly, to assist a female sibling.

*I give my siblings because I help my Mom in giving them pocket money to use for school. I want her to stop for a while in giving them money. If I have money, I give them to use at school.* (Participant 5956, 19 years, Interview 1/1)

Sharing money with sisters appeared to be more common than sharing with same age male siblings, and these relationships were reciprocal. One young woman who enrolled in the study at age 14 and who lived with a very large three–generation extended family talked in every interview of sharing the money with her sisters, giving them each a quarter of her R100 payment. She noted: *‘Mostly it’s because I don’t need school materials and don’t need personal things, so that’s why I thought they also have to be happy with that money and buy what they need.’* In a later interview she talked about this being a reciprocal relationship with her sister and that she chooses to share with her *‘because sometimes when I don’t have money she does give me when she has it.’*

Sharing with friends was far less common – but also discussed by a small number of participants. One young woman mentioned that she often gave money to one of her close friends:

*Since they deposit R100, when I withdraw it I only manage to get R90. Sometimes I give my friend R50 or even that R90 when she needs it seriously.* (Participant 4959, 16 years, Interview 1/1)

In this instance she noted that bank charges reduce her monthly payments to ZAR90, but has willingly given a friend this whole amount when the need arises. From later in the discussion it seems however that this example of sharing was in the form of a loan, rather than an outright gift, as there appears to be an expectation of being paid back. In the qualitative interviews no young women reported sharing their portion of the cash transfer with boyfriends or sexual partners. This is in contrast to the quantitative survey data and is indicative of the small qualitative sample.

Over and above the mechanisms that young women employed to access the transfers, make decisions about spending and whether they share the money with others, access to a regular sum of money held significant social meaning for young women who enrolled in the study. Specifically, the money allowed participants to assume an adolescent identity that was rooted in both an enhanced independence from their families and securing a place within their peer networks.

### The meaning of money for adolescent women: Independence

Most young women discussed the importance of being able to make one’s own decisions about money and having the independence to spend their own money without having to ask for it from family. For many, this was a completely unique experience; indeed one young woman specifically noted that she *never receives the amount of money like that* and therefore was very conscious of the independence this provided her. Participants spoke particularly of appreciating that they controlled decision-making and spending with regard to this money. A 16 year old participant who lives in a poor household with her mother and sisters said:

*When I go around and maybe want something in the shop, when I have the Swa Koteka money I get in the shop and buy; not to come back and ask. I am able to say that I have access to my own money.* (Participant 7915, 17 years, Interview 2/3)

This was echoed by many other participants, such as a 19-year old who, when asked about how being part of the cash transfer study felt, stated *‘It feels great because no one controls it but me’* and a 20-year-old who said *‘I feel good because when I want something I do it for myself.’* Further, a 19-year-old woman mentioned that to have control of your own money, allowed for control and independence more broadly when she mentioned that ‘*If someone can give you money, he/she will always shout at you every day and night.’* Throughout this participant’s interviews she reports going hungry and there is a real sense that she feels herself (and other children in the family) are a burden to their household in terms of the cost of feeding them. Through her interview she implies that when you receive money from others you are under their control and that in contexts of poverty this breeds resentment.

This appreciation for independence was also bound with family relationships, particularly with mothers, given that so many households in this community are single-parent families. Overall, there was a sense from the young women that they recognized the financial burden their requirements and desires placed on their families. Their access to the cash transfer allowed them an independence from their parents, and reduced the financial demands they made on families. Many of the participants discussed appreciating the feeling of independence ‘*because I don’t trouble them*.’ Among many other examples of such statements, two women both 17- years-old at the time of interview, reported relief at no longer perceiving themselves to be a burden or ‘bothering’ their parents.

*I feel happy because before I got money from Swa Koteka I used to bother my mother all the time. I used to buy [sanitary] pads and it was difficult for me, sometimes I felt like a burden to her.* (Participant 5833, 17 years, Interview 1/3)

*I feel well because I stop bothering my Mom for asking money to braid my hair.* (Participant 2811, 17 years, Interview 1/1)

Indeed, many young women spoke about better relationships with their families, mothers in particular, since reducing their requests for money. One of the participants suggests that this is the case when she mentions that:

*I avoid bothering people asking for money. People might get tired, even parents, when you keep on asking for money. She [mother] gets bored sometimes.* (Participant 4959, 16 years, Interview 1/1)

Most young women discussed independence from their families, but there were some limited indications that access to the cash transfer might also increase independence from male sexual partners. Indeed, one of the young women in the qualitative cohort noted that ‘*this money is good. Even if you don’t have a boyfriend you can buy hair extensions and body lotion’*, implying that such items were generally out of reach of those young women without boyfriends providing for them. There were, however, no young women who specifically discussed their own enhanced independence from boyfriends in the interviews.

### The meaning of money for young women: Enhanced peer status and identity

Identity formation and peer group belonging were frequently discussed in interviews. In discussions about their future aspirations, the young women in our study almost universally discussed their desire to have better lives than their parents – symbolically signified through a career, access to their own money, having their own home and assisting their parents and community. This is exemplified by one young woman who said *‘So I want to be a person that owns herself and do what I want in my time.’* Another said that her future aspirations were to *‘finish my studies and get a job, and have my own possessions’* and a further woman who noted that she would like to be a mechanical engineer as *‘it is the job that I like in future and I will be able to have enough money to buy what I want.’* Even in the present moment, the role of identity and finding a place within the peer group was premised on access to consumer items. This is exemplified by one participant who said that she was pleased with her involvement in the study *‘because friends like superior things and I am able to buy it with the money from Swa Koteka.’* Identity and acceptance within the peer group appear to be largely determined by two main factors: cleanliness and having visible resources, signified through adequate school uniform or having pocket money to spend at school.

A number of young women discussed how peer acceptance was influenced by their personal appearance at school. Although tuition costs are free at all schools included in this project, school uniform costs remained a significant burden for families in the area. Young women also discussed their need to have cosmetics and toiletries that would ensure that they were not embarrassed by their personal hygiene at school. Two young women discussed how they had been teased and ridiculed at school because of the poor state of their clothing and how the cash transfer money had played a significant role in changing this behavior.

*So, we have a feeding scheme at school and l don’t have a problem with the food we eat, but l did not have a school uniform and they were laughing at me, they were pointing fingers at me. So the money that I am getting from Swa Koteka, I bought a school uniform. It boosted my self-esteem because children at school are no longer laughing at me.* (Participant 5833, 17 years, Interview 1/3)

*As you can see my family background, the time I was attending school, there was no one who could buy me a t-shirt, if my shoes were damaged or skirts for schooling, I was using Swa Koteka money to buy.* (Participant 5212, 19 years, Interview 1/1)

Other young women specifically discussed their personal grooming. When asked why she spends the cash transfer on cosmetics, one young woman explained *‘we want to be beautiful. We want to be clean.’* Others also talked about the importance of cleanliness. In particular, an 18 year old participant sharing a one roomed home with eight other family members remarked:

*I regarded this as the most important things to me because when I go to school I must be clean and smell nicely to everyone.* (Participant 4728, 18 years, Interview 1/3)

A further necessity for peer group belonging was having access to cash, generally called ‘pocket money’ at school. From young women’s discussion it seems that pocket money at school was less about hunger than it was about conspicuous consumption. For example, one of the participants noted that girls tend to share food at school, but that it was good to be seen to spend pocket money. This is exemplified in one young woman stating *‘there is no change [in our relationship] because we are able to do what we were doing in the past. Like if another learner didn’t carry food at school, we share with her. If I didn’t carry mine, she shares with me.’* A participant who enrolled in the study when she was 14 years old and who spoke specifically about using her cash transfer as pocket money, mentioned that the change in her circumstances engendered by receiving the cash transfer allowed her to feel more included at school.

*Sometimes I went to school without pocket money. . . There are great changes [since participating in the study] because my friends were having money most of the time and I felt like I am left out.* (Participant 8129, 15 years, Interview 1/3)

Pocket money at school appears to be an important signifier for young women of their families’ ability to provide them with something extra, and they frequently discussed the shame and embarrassment of not having access to cash for snacks while at school. Students at all schools in the study area are eligible for a government school nutrition program that provides a cooked meal each day. There are, however, also informal traders who gather at the school gates selling snacks and sweets, and it is the ability to access these items that appears to hold significance for the participants in our study.

## Discussion

This study is one of the first to document the spending patterns of young African women directly receiving cash transfers as part of HIV prevention efforts. Quantitative and qualitative data from the study show that young women were largely responsible for decisions about accessing, spending and saving the cash transfer. This is similar to findings from Malawi where a similar proportion (two-thirds) of young women reported controlling decisions about spending their portion of both conditional and unconditional cash transfers [25], but is counter to general perspectives on income in poor families, in which it is generally accepted that income is pooled within households [26]. We specifically asked young women about negative experiences associated with receipt of the cash transfer, but these were almost universally not reported. We were particularly interested in this issue given that Baird, de Hoop and Özler speculated that increased psychological distress in conditional cash transfer recipients in Malawi was related to perceived pressure of being an income earner in their household [27]. It is possible that higher socio-economic standards more generally in South Africa compared to Malawi, and the fact that young women in our study did not seem to be expected to use their cash transfer for household expenditure, contributed to this difference - this despite the fact that at baseline in our study, over 80% of young women reported that their family received the CSG, while approximately one-third reported food insecurity [18], indicating a high level of relative poverty for South Africa. Caregivers were most likely to report spending their portion of the cash transfer on household groceries and other household items, possibly addressing family shortfall without having to resort to using the young women’s portion of the cash transfer. Despite the focus of the intervention on HIV prevention, almost no participants reported spending the cash transfer on condoms. This is not completely surprising as the communities in which we worked have seven local clinics and 3 health centers where free condoms are available and limited geographical access to sexual and reproductive health services where payment is required. Young women overwhelmingly reported spending their portion of the cash transfer on items of ‘symbolic capital’ – the clothes and accruements of adolescent life most likely to invoke peer acceptance and belonging [28].

The value of cash transfers as a vehicle for young women’s independence and autonomy should not be underestimated in this environment. Research with adolescents in other contexts has shown that the source of adolescent income impacts the degree of independence it offers [12]. In Palan et al.’s study of French and American adolescents [12], income earned by adolescents was more likely to engender independence than income provided by the family. In our setting, opportunities for adolescents to access their own money are limited: poverty prevents many families from being able to make regular contributions to their adolescent children, and community poverty and limited infrastructure mean that casual and part-time employment opportunities are very limited. The cash transfers therefore filled an important gap in income potential for adolescent girls living in this community, and were particularly perceived to engender independence.

The very low level of negative outcomes reported qualitatively from women receiving the cash transfer in our study is in contradiction to evidence from other African countries. A study in Uganda which randomized adolescent women to two different treatment groups (savings account only vs savings account and safe space group meetings with education on reproductive health and financial planning) found that women who received the full intervention were less likely to report experiences of verbal harassment than those receiving just the financial component of the intervention [29]. The authors concluded that interventions with adolescent women should focus on building both financial and social capital. The SHAZ! microcredit program in Zimbabwe also reported negative consequences for young female recipients, although experiences of harassment and fear of theft were particularly related to small business ownership, the goal of the intervention microloans, rather than having access to cash [20].

Although there were concerns expressed during community and service provider meetings about the study in the MRC/Wits-Agincourt HDSS, and among stakeholders working in social protection globally about the consequences of allowing adolescent women access to cash, our findings suggest very little spending on items such as cigarettes and alcohol. These findings are similar to evidence from a number of other cash transfer programs for adolescents in other regions of the world. *Yo Puedo* (“I can” in Spanish), a cash transfer program for adolescents aged 16–21 years in San Francisco, similarly found no evidence that program participants used their cash payments for illicit or high-risk behaviors. Indeed, despite their intervention not specifically focusing on substance use, they noted that youth receiving cash were less likely to report spending on alcohol and marijuana [30]. The Mexican *Oportunidades* (“Oportunities” in Spanish) program for male and female adolescents also did not note negative consequences; in fact, the *Oportunidades* program noted a reduction in smoking and alcohol consumption associated with access to social grants [31]. Our study adds an African perspective to this issue.

Although our cash transfer was premised on assisting families with the costs of education, the reality in South Africa and many sub-Saharan African countries is that (primary) education is free (including secondary in South Africa) and therefore additional money in households can be used to symbolically distance adolescents from poverty and enhance their standing in peer networks. This was certainly the case in our study where young women mentioned that being able to buy new school uniforms and access to ‘pocket money’ allowed them to feel a greater sense of belonging with their peers. An intervention in Zimbabwe noted similar findings. In this study, the impacts of a community-led cash transfer intervention were viewed as increasing school attendance, but also allowing school-going children to ‘look smart’ [32].

Largely driven by concerns about the role of transactional sex in increasing young sub-Saharan African women’s HIV risk, there is an emerging body of literature concerned with what has been called the ‘commodities of modernity’ [10]. In initial discussions of adolescent transactional sex, the focus was on the role of poverty and young women’s desperate attempts to access food and shelter. Increasingly though, has been the recognition that *perception* of need is significant and that young women’s needs have increasingly come to include ‘commodities that are symbolic of sophistication and a lifestyle commonly perceived as modern’ [10, p.224], also sometimes called ‘symbol capital’ [33]. A focus on symbol capital has been recognized in post-apartheid changes to South Africa, in which worth is increasingly measured through consumerism and self-esteem determined by peer-pressure to achieve material goods [26]. Adolescents have very limited access to real power; however, consumerism allows for access to status power within the peer group and is, therefore, a significant motivator in developing adolescent identity [11]. It has been noted that “All teenagers, including those living in low-income environments, have been affected by the cultural focus on wealth, within which money equals power and consumption marks independence and defines the self” [11, p.232]. Certainly, our results show that young women in this community are acutely aware of the symbolic value of their appearance to status in the peer network and perceived social attributes. The cash transfer represents a significant resource for young women to participate in a ‘modern’ lifestyle in the context of limited family income and resources.

Deutsch and Theodorou [11] also note that adolescents tend to minimize the economic gap by presenting an image of higher socio-economic status than they actually have; one which is not tempered by their actual family socio-economic status. Certainly, responses to questions about their future aspirations suggest that our participants’ *perception* of their need for items beyond their parents’ economic means is key to development of their autonomy and social standing. The fact that the majority of our participants chose to spend their cash transfer on toiletries and cosmetics is in keeping with previous researchers’ findings that young women “engage in consumptive acts which solidify and reinforce their present engendered identities as feminine while constructing future aspirations aimed at securing individual well-being as financially independent adults” [11, p. 245]. This was particularly true for older participants in our study, among whom a greater proportion reported spending the cash transfer on items to enhance their personal appearance. Perhaps this is a reflection of the relatively limited opportunities for independence that exist for young women once they leave school in this community.

## Conclusions

The findings from this study show that providing cash transfers to young women living in low socio-economic communities was largely associated with positive outcomes. This is despite the lack of impact on the intended outcomes of HIV infection and pregnancy reduction. Access to money, independent of their parents, is a key developmental issue for adolescents both in terms of their emerging autonomy and developing identity. While these issues may not be the key rationale for social protection programs, our findings illustrate additional unplanned benefits of such programs for adolescent women. These findings are significant at a time when increasing pressure is being felt to address structural drivers of HIV risk, and for female adolescents in particular. The results of this study show that adolescents themselves can be the recipients of social grants without the concerns about negative outcomes related to their vulnerable status as young people being realized.

## Abbreviations

ACASI: Audio Computer Assisted Self Interview
ATM: Automated Teller Machine
CSG: Child Support Grant
HDSS: Health and Socio-Demographic Surveillance System
HIV: Human Immunodeficiency Virus
HSV-2: Herpes Simplex Virus Type 2
ICR: Intercoder Reliability
ZAR: South African Rand (also abbreviated as R100)

## Footnotes

1 Reflects age at the time of the interview. Interview number of total qualitative interviews
